# Making Medicare Complicated: The Consequences of Privatization

**DOI:** 10.1093/geroni/igab046.971

**Published:** 2021-12-17

**Authors:** Pamela Herd

**Affiliations:** Georgetown University, Georgetown University, District of Columbia, United States

## Abstract

Starting with policy changes in the 1980s, Medicare has largely become privatized, with nearly 40 percent of beneficiaries enrolled in private Medicare Advantage plans and another 30 percent with private supplemental coverage, including for prescription drug coverage. As a result, Medicare has become laden with administrative burdens and barriers. Beneficiaries are faced with a confusing array of plans and coverage options when they enroll, and are expected to choose a new plan every year. The choice they make has large implications for their health care costs, as well as their actual access to health care. While we typically think that targeted policies are burdensome and social insurance programs are accessible, Medicare contradicts this easy categorization. Instead, it demonstrates how private sector involvement in public programs can increase complexity and increase burdens for beneficiaries.

